# Predict potential pharmacological mechanisms of Ling-gui-Zhu-gan Decoction in treating unstable angina pectoris using liquid chromatography-mass spectrometry and network pharmacology

**DOI:** 10.3389/fchem.2025.1649538

**Published:** 2025-08-04

**Authors:** Xiaopeng Li, Tan Xue, Panpan Zhang, Fengyu Dong, Han Li, Jing Yao, Haiying Huang, Lizhuang Zhang, Ruixin Liu

**Affiliations:** ^1^ Department of Pharmacy, First Affiliated Hospital of Henan University of Traditional Chinese Medicine, Zhengzhou, Henan, China; ^2^ Henan University of Traditional Chinese Medicine College of Pharmacy, Zhengzhou, Henan, China; ^3^ Traditional Chinese Medicine Modernization School Enterprise R & D Center, Henan University of Traditional Chinese Medicine, Henan Tailong Pharmaceutical Co., Ltd., Zhengzhou, Henan, China; ^4^ Henan Traditional Chinese Medicine Clinical Application, Evaluation and Transformation Engineering Research Center, Zhengzhou, Henan, China; ^5^ Henan Provincial Key Laboratory of Clinical Pharmacy of Traditional Chinese Medicine, Zhengzhou, Henan, China; ^6^ Henan University of Traditional Chinese Medicine Respiratory Disease Prevention and Treatment of Traditional Chinese Medicine to Build Collaborative Innovation Center, Zhengzhou, Henan, China; ^7^ Henan Institute of Chinese Medicine, Henan University of Traditional Chinese Medicine, Zhengzhou, Henan, China; ^8^ Engineering Research Center of Ministry of Education, Pharmaceutical and New Drug Development of Traditional Chinese Medicine, Beijing, China

**Keywords:** Ling-Gui-Zhu-Gan Decoction, transfer rate, UHPLC-Q-Orbitrap/MS, unstable angina, network pharmacology

## Abstract

**Introduction:**

Ling-Gui-Zhu-Gan Decoction (LGZGD), one of the first batches of classical Chinese prescriptions formally recognized by the Chinese government, has a long-standing history of clinical application and significant potential for modern development. However, the chemical composition and content of different types of pharmaceutical preparations are not clear.

**Methods:**

This study aimed to develop an analytical approach integrating HPLC and UHPLC-Q-Orbitrap/MS to comprehensively characterize the chemical constituents of LGZGD across different preparation stages and to investigate its pharmacodynamic basis in the treatment of unstable angina pectoris (UA) using network pharmacology. The content and transfer rate of six index components were quantified using HPLC.

**Results:**

A total of 75 compounds were identified via UHPLC-Q-Orbitrap/MS, comprising 24 flavonoids, 25 organic acids, nine phenylpropanoids, eight terpenoids, five saponins, and four other compounds, based on precursor ion peaks and fragment ion spectra. Notably, five compounds—(-)-pterocarpin glucoside, γ-aminobutyric acid, calycosin, trimethyl citrate, and proline-phenylalanine—were absent following the drying of the concentrate. Using the LC-MS data as a foundation, network pharmacology and molecular docking analyses were conducted to elucidate the pharmacodynamic components responsible for LGZGD’s therapeutic effects on UA. This integrative analysis identified three key active compounds—naringenin, glycyrrhizin, and calycosin—and three core targets: TNF, EGFR, and PTGS2.

**Discussion:**

The analytical method established in this study effectively delineates the chemical profile and index component transfer dynamics of LGZGD preparation intermediates, providing essential data for the development of both liquid and solid dosage forms. The constructed “medicine-component-target-pathway-disease” network preliminarily reveals the multi-component, multi-target, and multi-pathway mechanisms by which LGZGD may exert therapeutic effects on UA. This work provides a scientific foundation for its clinical application, supporting rational drug use and formulation development.

## 1 Introduction

Traditional Chinese medicine (TCM), as a distinctive medical system, has garnered increasing recognition both within China and internationally. Classical prescriptions refer to formulas documented in medical texts from the Qing Dynasty or earlier that continue to be widely used due to their well-established efficacy and distinct clinical advantages (Medicine National Administration of Traditional Chinese, 2016). To accelerate the development and modernization of such formulations, the National Administration of Traditional Chinese Medicine has released over 300 classical prescriptions in two successive batches (Medicine National Administration of Traditional Chinese, 2018; Medicine National Administration of Traditional Chinese, 2023).

Historically, the development of TCM-based new drugs has followed a research paradigm akin to that of Western pharmaceuticals, emphasizing data from pharmaceutical, non-clinical, and clinical studies to ensure safety, efficacy, and consistent quality for regulatory approval. Classical prescriptions represent the culmination of centuries of empirical clinical practice and are generally regarded as safe and effective.

Ling-Gui-Zhu-Gan Decoction (LGZGD), included in the first batch of officially recognized classical prescriptions, was originally documented in Zhang Zhongjing’s “Treatise on Febrile Diseases” during the Eastern Han Dynasty. It has been applied in the treatment of various conditions, including chronic heart failure (Zhou and Hu, 2021), angina pectoris (Su et al., 2017), malignant pleural effusion (He et al., 2016), cirrhotic ascites (Kong and Liu, 2021), non-alcoholic fatty liver disease (Dong et al., 2022), and posterior circulation ischemic vertigo (Cai et al., 2024). The decoction comprises four medicinal materials: *Fu-ling* (*Poria cocos*), *Gui-zhi* (*Cinnamomum cassia*), *Bai-zhu* (*Atractylodes macrocephala*), and *Gan-cao* (*Glycyrrhiza* spp.).

*Fu-ling* is the dried sclerotium of *P. cocos*, rich in triterpenes and polysaccharides. *Gui-zhi*, the dried twig of *C. cassia*, primarily contains volatile oils and organic acids, with cinnamaldehyde comprising 70%–80% of the volatile fraction. The predominant organic acid is cinnamic acid. *Bai-zhu*, derived from the dried rhizome of *A. macrocephala*, contains volatile oils, lactones, and organic acids, among which chlorogenic acid is a key component. *Gan-cao* includes the dried roots and rhizomes of *Glycyrrhiza uralensis*, *Glycyrrhiza inflata*, or *Glycyrrhiza glabra*, and is known for its saponins (notably glycyrrhizic acid and liquiritin), flavonoids, and polysaccharides.

Due to the complexity of TCM formulations, chemical composition can vary significantly depending on the preparation process. While recent research has focused primarily on quantifying index components and their transfer rates during preparation, broader chemical profiling remains limited. At present, the existing studies on the HPLC transfer rate of LGZGD index components mainly focus on the chemical components of *Gan-cao* and *Gui-zhi*. In this study, the index components of *Bai-zhu* were added on this basis. Moreover, no studies to date have applied network pharmacology to investigate LGZGD’s mechanisms in treating unstable angina pectoris (UA).

To address these gaps, this study selected LGZGD as the research subject and UA—an unexamined indication—as the disease target. We employed UHPLC-Q-Orbitrap/MS to profile the chemical constituents across LGZGD’s preparation intermediates, alongside HPLC analysis to quantify six key index components and assess their transfer rates. The integrated results were then subjected to network pharmacology analysis to predict LGZGD’s pharmacodynamic basis in UA therapy. This work lays the groundwork for the mechanistic understanding, formulation optimization, and evidence-based clinical application of LGZGD.

## 2 Experimental methods

### 2.1 Apparatus and reagents

#### 2.1.1 Apparatus

The following instruments were employed in this study: an Agilent 1,260 high-performance liquid chromatograph (HPLC); a Waters xSelect HSS T3 chromatographic column (4.6 mm × 250 mm, 5 μm); a ceramic decoction pot (Model: 30MF53L, Shenzhen Zhengyun Technology Co., Ltd.); a Thermo Scientific Orbitrap Exploris 240 high-resolution LC-MS system; a rotary evaporator (Model: RE-52AA, Shanghai Yarong Biochemical Instrument Factory); a Hypersil GOLD™ column (100 × 2.1 mm, 1.9 μm); a PL-FS80T ultrasonic cleaner (Dongguan, China); a B-290 spray dryer (BUCHI, Switzerland); and a 0.01 mg precision electronic balance (Sartorius, Göttingen, Germany).

#### 2.1.2 Reagents

Methanol and ethanol were obtained from Yongda (Tianjin, China). Chromatography-grade acetonitrile, phosphoric acid, and formic acid were purchased from Merck (Darmstadt, Germany). Analytical standards—cinnamic acid, cinnamaldehyde, neochlorogenic acid, cryptochlorogenic acid, liquiritin, and glycyrrhizic acid (each with purity >98%)—were supplied by Yuanye (Shanghai, China). All crude drug samples were sourced from various medical institutions and traditional decoction piece markets across China. Samples were sequentially numbered within each category and randomly combined to form drug pair groups.

### 2.2 Sample preparation

#### 2.2.1 Medicinal herbal pieces (MHP)

Each herbal sample was pulverized and passed through a 50-mesh sieve.

Guizhi powder (0.5 g) was placed in a conical flask, extracted with 25 mL of pure methanol via ultrasonic treatment for 10 min (350 W, 35 kHz), allowed to stand for 24 h, then ultrasonically extracted again under the same conditions. After cooling, the solution was weighed, and methanol was added to restore the original weight. The extract was filtered through a 0.45 μm microporous membrane. Then, 0.1 mL of the filtrate was accurately transferred to a 25 mL volumetric flask and diluted to volume with pure methanol to yield the Guizhi sample.

A 1.0 g sample of Baizhu powder was extracted with 60 mL of 80% (v/v) methanol via ultrasonic treatment for 10 min (500 W, 40 kHz). The solution was brought back to original volume with 80% methanol, filtered through a 0.45 μm microporous membrane to obtain the Baizhu sample.

A 0.1 g sample of Gancao powder was extracted with 50 mL of 70% (v/v) ethanol via ultrasonic treatment for 30 min (250 W, 40 kHz). After compensating for volume loss with 70% ethanol, the extract was filtered using a 0.45 μm membrane to yield the Gancao sample.

#### 2.2.2 LGZGD water decoction (WD)

A total of 62.5 g Fuling, 46.9 g Guizhi, 46.9 g Baizhu, and 31.3 g Gancao were placed in a ceramic pot and soaked in 1,200 mL of water for 30 min. The mixture was initially boiled at 1800 W, then decocted at 700 W for 2 h. The resulting decoction was diluted to 600 mL, further diluted to 10 mL with 50% (v/v) methanol, ultrasonicated for 15 min, and centrifuged at high speed for 10 min to prepare the water decoction sample.

#### 2.2.3 LGZGD concentrated solution (CS)

A 300 mL portion of the water decoction was concentrated using a rotary evaporator at 60 °C and 50 rpm under reduced pressure (−0.06 to −0.08 MPa) to a final volume of 60 mL. From this concentrate, 2 mL was diluted to 10 mL with distilled water. Subsequently, 5 mL of this solution was diluted to 10 mL with 50% (v/v) methanol, ultrasonicated for 15 min, and centrifuged for 10 min to obtain the concentrated solution sample.

#### 2.2.4 LGZGD spray-dried powder (SDP)

The concentrated decoction was heated to 60°C using an electromagnetic stirrer. Spray-drying was performed at an inlet air temperature of 120°C and an outlet air temperature of 90°C–100°C. After collection, 0.5 g of the spray-dried powder was diluted to 10 mL with distilled water and ultrasonicated for 30 min. Once fully dissolved, 5 mL of this solution was diluted to 10 mL with 50% (v/v) methanol, ultrasonicated again for 15 min, and then centrifuged at high speed for 10 min to yield the spray-dried sample.

### 2.3 Reference standard solutions

Accurately weighed amounts of cinnamic acid, cinnamaldehyde, neochlorogenic acid, cryptochlorogenic acid, liquiritin, and glycyrrhizic acid were each dissolved in methanol to prepare individual standard solutions with concentrations of 0.032, 0.016, 0.063, 0.022, 0.083, and 0.395 mg.mL^-1^, respectively.

### 2.4 Quantification and transfer rate analysis of LGZGD index components

#### 2.4.1 Chromatographic conditions

Chromatographic separation was performed using a Waters XSelect HSS T3 column (4.6 mm × 250 mm, 5 μm). The mobile phase consisted of acetonitrile (A) and 0.1% phosphoric acid in water (B) under the following gradient program: 0–20 min, 90%–85% B; 20–30 min, 85%–82.5% B; 30–35 min, 82.5% B; 35–50 min, 82.5%–75% B; 50–70 min, 75%–65% B; 70–80 min, 65% B; 80–81 min, 65%–95% B; 81–96 min, 95% B. The flow rate was 1.0 mL/min, with the column maintained at 30°C. Injection volume was 10 μL. Detection wavelengths were as follows: cinnamic acid at 275 nm; cinnamaldehyde at 290 nm; neochlorogenic and cryptochlorogenic acids at 327 nm; liquiritin at 230 nm; and glycyrrhizic acid at 254 nm.

#### 2.4.2 Method validation

To assess linearity, standard solutions were serially diluted, and peak areas were recorded. Calibration curves were constructed by plotting mass concentration (μg·mL^-1^, X-axis) against peak area (Y-axis). Regression equations, *R* ^2^ values, and linear ranges were calculated for each compound.

Precision was evaluated by injecting one water decoction sample six consecutive times. Repeatability was assessed by independently preparing and analyzing six decoction samples. Stability was tested by analyzing the same decoction sample at 0, 2, 4, 6, 8, 12, 18, and 24 h. The relative standard deviation (RSD) of each compound’s peak area, using the corresponding reference peak as a standard, was calculated.

For recovery testing, known quantities of the analytes (0.079 mg cinnamic acid, 0.128 mg cinnamaldehyde, 0.008 mg neochlorogenic acid, 0.007 mg cryptochlorogenic acid, 0.786 mg liquiritin, and 0.928 mg glycyrrhizic acid) were added to six half-concentration decoction samples. Recovery rates and RSD values were determined under the same chromatographic conditions.

### 2.5 Analysis of LGZGD chemical constituents

#### 2.5.1 Chromatographic conditions

Chromatographic separation was performed using a Hypersil GOLD™ column (100 × 2.1 mm, 1.9 μm). A gradient elution was carried out with 0.1% formic acid in water (mobile phase A) and methanol (mobile phase B) under the following conditions: 0–4 min, 2%–10% B; 4–8 min, 10%–25% B; 8–11 min, 25%–45% B; 11–15 min, 45%–50% B; 15–20 min, 50%–70% B; 20–22 min, 70%–90% B; 22–27 min, 90%–95% B. The flow rate was set at 0.2 mL/min, with the column temperature maintained at 25°C. The injection volume was 10 μL.

#### 2.5.2 Mass spectrometry conditions

Mass spectrometric detection was performed using a heated electrospray ionization (H-ESI) source. The spray voltages were set to 3500 V in positive ion mode and 3000 V in negative ion mode. The ion transfer tube temperature was 350°C, and the auxiliary heater was maintained at 325°C. Sheath and auxiliary gas flow rates were set to 35 arb and 12 arb, respectively. Data were acquired in full scan mode (MS^1^) and data-dependent MS/MS mode (dd- MS^2^). The MS^1^ resolution was 120,000 over an m/z range of 70–1,050, with an RF level of 70%. MS^2^ spectra were acquired with a resolution of 15,000 using auto-selected ion scanning ranges and normalized collision energies of 20%, 40%, and 60%.

#### 2.5.3 Identification of LGZGD constituents

Water decoction, concentrated extract, and spray-dried powder forms of LGZGD were prepared and analyzed. Total ion current (TIC) chromatograms were recorded in both positive and negative ion modes. Data analysis was performed using Thermo Xcalibur 2.1. Accurate precursor ions (*m/z*) were identified via MS^1^, and corresponding fragment ions were obtained from MS^2^ spectra. Component identification was confirmed by comparing MS^2^ fragmentation patterns with those reported in the literature and available databases.

### 2.6 Network pharmacology-based prediction of LGZGD active components for UA treatment

#### 2.6.1 Construction of compound and disease target databases

Chemical constituents identified in LGZGD via LC-MS were further screened using the TCMSP database, focusing on Poria cocos, Ramulus cinnamomi, Atractylodes macrocephala, and Glycyrrhiza uralensis. Compounds with oral bioavailability (OB) > 30% and drug-likeness (DL) > 0.15 that were also detected by LC-MS were selected as potential bioactive ingredients.

#### 2.6.2 Protein-protein interaction (PPI) network analysis

The intersecting targets of LGZGD and unstable angina (UA)-related pathways were entered into the STRING v12.0 database (species: *Homo sapiens*), with the interaction confidence threshold set to “medium” (0.4). Output data were exported in TSV format and imported into Cytoscape 3.9.1 for network visualization. Node size and color depth represented the degree of centrality. Nodes with degrees exceeding twice the median were initially filtered. Secondary screening for Degree, BC, CC > median targets. The top five hub targets were selected for molecular docking analysis.

#### 2.6.3 GO and KEGG pathway enrichment analysis

Intersection targets were submitted to the DAVID v6.8 database (species: *H. sapiens*) for Gene Ontology (GO) and Kyoto Encyclopedia of Genes and Genomes (KEGG) enrichment analyses. GO terms were categorized into cellular component (CC), biological process (BP), and molecular function (MF). Results were ranked by ascending *p*-values, and the top 10 GO terms and top 20 KEGG pathways were visualized in bubble plots.

#### 2.6.4 Construction of the “medicine–component–target–pathway–disease” network

KEGG-enriched pathways were integrated with intersection targets and visualized using Cytoscape 3.9.1 to construct a comprehensive “medicine–component–target–pathway–disease” network. Network topology parameters were calculated, and the top five key active ingredients were identified based on degree values.

#### 2.6.5 Molecular docking verification

The five core targets from Section 2.6.2 and the five key active ingredients from Section 2.6.4 underwent pairwise molecular docking (25 combinations). Binding affinities were used to evaluate docking stability and reliability. Three-dimensional structures of core targets were retrieved in PDB format from the UniProt database, while compound structures in SDF format were downloaded from PubChem and converted to PDB format using Open Babel. Ligands and water molecules were removed using PyMOL. Grid boxes were defined in AutoDockTools based on the spatial configuration of target proteins. Docking simulations were conducted, and the results were visualized using PyMOL.

## 3 Results

### 3.1 Analysis of marker compound transfer rates in LGZGD

#### 3.1.1 Method validation

The calibration curves for all marker compounds demonstrated excellent linearity within their respective concentration ranges, with *R* ^2^ exceeding 0.999 (Supplementary Table S1). Methodological validation results, including precision, repeatability, stability, and recovery, are summarized in Supplementary Table S2. The results confirmed that the analytical system exhibited high precision and repeatability, with good sample stability over a 24-h period. Recovery rates also met acceptable analytical standards.

#### 3.1.2 Confirmation of marker components

Chemical fingerprinting was performed using ChemPattern™ software. Representative chromatograms at 275 nm are shown in Supplementary Figure S1 and Supplementary Figure S2. Sample similarity analysis based on the common pattern mode yielded similarity indices >0.94, indicating good batch-to-batch consistency and overall quality stability of LGZGD samples.

#### 3.1.3 Transfer rates of marker components

Transfer rates for all six marker compounds across the decoction pieces, decoction, concentrated solution, and spray-dried powder solution are presented in Table 1. Cinnamaldehyde exhibited a particularly low transfer rate—from decoction pieces to decoction (1.01%) and from decoction to concentrated solution (6.79%). In contrast, cinnamic acid, neochlorogenic acid, cryptochlorogenic acid, liquiritin, and glycyrrhizic acid showed more stable transfer rates across preparation stages, supporting their utility as robust quality control indicators for LGZGD.

**TABLE 1:** Concentration and transfer rates of marker compounds.

Index component	Content (mg·g^-1^) and transfer rate							
	MHP	Rate	WD	Rate	CS	Rate	SDP	Total rate
Liquiritin	14.662	43.51%	6.331	72.04%	4.527	77.60%	3.451	23.54%
Glycyrrhizic acid	30.357	25.53%	7.583	79.47%	6.008	82.70%	4.921	16.21%
Cinnamic acid	1.450	49.49%	0.716	80.19%	0.577	78.01%	0.449	30.97%
Cinnamaldehyde	16.562	1.01%	0.165	6.79%	0.008	47.11%	0.004	0.02%
Neochlorogenic acid	0.064	70.65%	0.058	80.21%	0.038	80.37%	0.020	31.25%
Cryptochlorogenic acid	0.057	62.75%	0.034	77.27%	0.025	83.32%	0.020	35.09%

Note: Transfer rate = (Sample volume × component concentration in solution)/mass of decoction pieces.

Cinnamaldehyde is the principal volatile constituent of *Ramulus cinnamomi*, accounting for approximately 87% of its essential oil content (Li et al., 2024a). Despite reduced-pressure conditions during concentration, cinnamaldehyde, owing to its high volatility and thermal instability, suffers substantial loss (>90%). The spray-drying process, involving brief exposure to high temperatures, further exacerbates its degradation, thus explaining the low overall transfer rate.

### 3.2 Identification of chemical constituents

**FIGURE 1:**
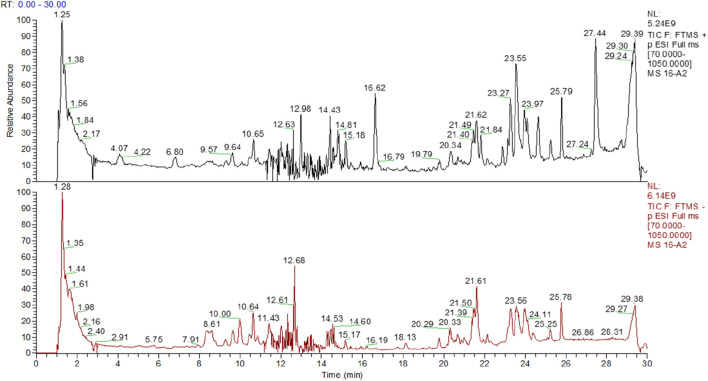
TICs of LGZGD samples in positive and negative ion modes.

Using UHPLC-Q-Orbitrap/MS, we identified the chemical constituents of LGZGD in its decoction, concentrated solution, and spray-dried powder forms. Total ion chromatograms (TICs) in both positive and negative ion modes are shown in Figure 1A total of 75 compounds were identified, comprising 24 flavonoids, 25 organic acids, nine phenylpropanoids, eight terpenoids, five saponins, and four miscellaneous compounds (Table 2).

**TABLE 2:** Identified components of LGZGD by UHPLC-Q-Orbitrap/MS.

NO.	tR/min	Molecular formula	Ion mode	Theoretical (m/z)	Actual (m/z)	δ (ppm)	Main fragment ion MS/MS (m/z)	Compounds	Type
1	21.61	C_22_H_24_O_9_	[M + H]^+^	433.1493	433.1491	−0.46	403.1023; 418.1257	(-)-medipterocarpin glucoside (Wu et al., 2024)	F
2	14.05	C_21_H_22_O_10_	[M-H]^-^	433.1129	433.1133	0.75	271.0611; 151.0037	5-Hydroxyglycyrrhizin (Li et al., 2011)	F
3	1.76	C_6_H_6_O_3_	[M + H]^+^	127.0390	127.0388	−1.66	109.0282; 81.0333; 69.0334	5-hydroxymethyl furaldehyde (Wang et al., 2024)	OA
4	20.466	C_18_H_22_O_6_	[M-H]^-^	333.1333	333.1344	3.32	305.1375; 261.1492	6-(3-hydroxy-propionyloxy) atractylenolide III (Lin et al., 2024)	T
5	21.83	C_16_H_22_O_3_	[M + H]^+^	263.1642	263.1642	0.19		8β-methoxy atractylenolide I (Liu et al., 2021)	T
6	6.67	C_11_H_12_N_2_O_2_	[M + H]^+^	205.0972	205.0969	−1.14	188.0705; 146.0600; 118.0650	D-tryptophan (Wang et al., 2024)	OA
7	1.96	C_6_H_14_N_4_O_2_	[M + H]^+^	175.1190	175.1190	0.04	158.0922; 130.0974; 116.0705	L-arginine (Yang et al., 2024)	OA
8	2.07	C_9_H_11_NO_3_	[M + H]^+^	182.0812	182.0812	0.22	165.0545; 147.0438; 136.0755	L-tyrosine (Wang et al., 2024)	OA
9	2.09	C_6_H_13_NO_2_	[M + H]^+^	132.1019	132.1019	−0.19	86.0963; 69.0698; 56.0499	L-isoleucine (Yang et al., 2024)	OA
10	1.26	C_4_H_9_NO_2_	[M + H]^+^	104.0706	104.0715	8.41	87.0440; 60.0807; 45.0334	γ-Aminobutyric acid (Wang et al., 2024)	OA
11	21.84	C_15_H_18_O_2_	[M + H]^+^	231.1380	231.1379	−0.29	203.0854; 163.0753; 119.0855	Atractylenolide I (Li et al., 2023)	T
12	22.83	C_15_H_20_O_2_	[M + H]^+^	233.1536	233.1537	0.40	215.1431; 187.1481; 131.0854	Atractylenolide II (Li et al., 2023)	T
13	4.18	C_9_H_11_NO_2_	[M + H]^+^	166.0863	166.0861	−1.12	131.0492; 120.0807; 103.0541	Phenylalanine (Wang et al., 2024)	OA
14	10.62	C_15_H_14_O_6_	[M-H]^-^	289.0707	289.0702	−1.75	137.0237; 123.0444; 109.0289	(+)-Epicatechin (L et al., 2021)	O
15	19.45	C_16_H_14_O_4_	[M + H]^+^	271.0965	271.0965	0.16	177.0545; 107.0489	Licorice chalcone (Hou et al., 2023)	F
16	2.14	C_4_H_6_O_4_	[M-H]^-^	117.0182	117.0194	9.78	73.0295	succinic acid (Li et al., 2024b)	OA
17	11.55	C_9_H_10_O_4_	[M + H]^+^	183.0652	183.0651	−0.74	155.0702; 140.0468; 123.0439	Syringaldehyde (Yin et al., 2024)	F
18	11.99	C_10_H_8_O_4_	[M + H]^+^	193.0495	193.0495	−0.34	178.0259; 150.0312; 133.0283	Scopoletin (Liu et al., 2024a)	P
19	13.72	C_9_H_8_O_3_	[M-H]^-^	163.0390	163.0401	6.80	119.0502	P-coumaric acid (Liu et al., 2024b)	OA
20	5.67	C_6_H_6_O_2_	[M-H]^-^	109.0284	109.0294	9.48	81.0345; 67.5919	Catechol (Liu et al., 2023a)	OA
21	14.85	C_9_H_8_O	[M + H]^+^	133.0648	133.0647	−1.06	115.0541; 103.0542	Trans-cinnamaldehyde (Xiao et al., 2024)	O
22	15.19	C_16_H_20_O	[M + H]^+^	229.1587	229.1569	−7.91	214.0987; 173.1325; 133.1012	Furan sesquiterpenes (Yang et al., 2024)	T
23	20.35	C_30_H_44_O_5_	[M + H]^+^	485.3262	485.3258	−0.70	467.3151; 449.3050; 431.2907	Pachymic acid B (Wang et al., 2024)	T
24	21.38	C_32_H_48_O_6_	[M + H]^+^	529.3524	529.3526	0.44	451.3211; 119.0855; 107.0854	Pachymic acid DM (Wang et al., 2024)	T
25	2.08	C_4_H_4_O_4_	[M-H]^-^	115.0026	115.0035	8.30	71.0140	Fumaric acid (Wang et al., 2024)	OA
26	14.82	C_16_H_14_O_5_	[M-H]^-^	285.0758	285.0777	6.84	270.0535; 149.0246	Licochalcone B (Yin et al., 2024)	F
27	12.24	C_21_H_22_O_9_	[M-H]^-^	417.1180	417.1195	3.60	255.0662; 135.0088	Liquiritin (Sun et al., 2023)	F
28	16.30	C_36_H_38_O_16_	[M-H]^-^	725.2076	725.2089	1.80	549.1615; 531.1507; 255.0662	Liquiritin A (Yin et al., 2024)	F
29	23.00	C_22_H_22_O_6_	[M-H]^-^	381.1333	381.1342	2.35	366.1111; 351.0874; 323.0553	Glycyrin (Yu et al., 2023)	F
30	12.64	C_15_H_12_O_4_	[M-H]^-^	255.0652	255.0664	4.84	153.0195; 135.0089; 119.0503	Liquiritigenin (Mengying et al., 2024)	F
31	23.96	C_42_H_62_O_16_	[M-H]^-^	821.3954	821.3958	0.41	803.3861; 759.3911; 645.3616	Glycyrrhizic acid (Mengying et al., 2024)	S
32	23.92	C_42_H_64_O_15_	[M-H]^-^	807.4161	807.4166	0.60	351.0566; 193.0353	Glycyrrhizin B2 (Wang et al., 2013)	S
33	23.39	C_42_H_62_O_17_	[M-H]^-^	837.3903	837.3903	−0.08	351.0567; 289.0543	Glycyrrhizin G2 (Tao et al., 2013)	S
34	25.96	C_42_H_62_O_16_	[M-H]^-^	821.3954	821.3969	1.82	351.0569; 193.0351; 113.0246	Glycyrrhizin H2 (Wang et al., 2016)	S
35	21.98	C_42_H_64_O_16_	[M-H]^-^	823.4111	823.4109	−0.21	351.0567; 193.0353	Glycyrrhizin J2 (Montoro et al., 2011)	S
36	13.80	C_16_H_12_O_6_	[M + H]^+^	301.0707	301.0704	−0.88	286.0472	Hispidulin (Xiao et al., 2024)	F
37	20.97	C_18_H_16_O_4_	[M-H]^-^	295.0965	295.0980	5.00	277.0871; 233.0973; 205.1024	Truxinic acid (Yin et al., 2024)	OA
38	21.60	C_30_H_44_O_4_	[M + H]^+^	469.3312	469.3311	−0.31	451.3202; 405.3149	Glycyrrhizic ester (Yin et al., 2024)	T
39	12.24	C_15_H_12_O_7_	[M-H]^-^	303.0499	303.0487	−3.92	285.0404; 177.0194; 125.0244	(-)-taxifolin (Du et al., 2024)	F
40	14.23	C_27_H_30_O_13_	[M-H]^-^	561.1603	561.1605	0.45	267.0661; 252.0426	Glycyroside (Lin et al., 2023)	F
41	10.16	C_9_H_8_O_4_	[M-H]^-^	179.0339	179.0351	6.84	135.0452	Caffeic acid (Yang et al., 2024)	OA
42	13.81	C_20_H_26_O_6_	[M-H]^-^	361.1646	361.1658	3.50	165.0556; 121.0295	Secoisolarciresinol (Chen et al., 2020)	P
43	4.02	C_7_H_12_O_6_	[M-H]^-^	191.0550	191.0559	4.37	129.0200; 111.0088; 87.0087	Quinic acid (Xiao et al., 2022)	OA
44	2.24	C_11_H_20_N_2_O_5_	[M + H]^+^	261.1445	261.1450	2.00	148.0603; 130.0498; 86.0963	Leucine-Glutamic acid (Wang et al., 2024)	OA
45	6.47	C_11_H_21_N_3_O_4_	[M + H]^+^	260.1605	260.1605	0.03	130.0500; 86.0963; 84.0807	Leucine-Glutamine (Wang et al., 2024)	OA
46	14.44	C_22_H_22_O_9_	[M + H]^+^	431.1337	431.1348	2.65	269.0806; 254.0578	Ononin (Xu et al., 2021)	F
47	16.19	C_16_H_12_O_4_	[M-H]^-^	267.0652	267.0663	3.99	252.0427; 223.0400	Onocol (Zhu et al., 2022)	F
48	15.65	C_16_H_12_O_5_	[M + H]^+^	285.0758	285.0760	0.95	270.0524; 253.0496; 225.0545	Calycosin (Han et al., 2007)	F
49	2.09	C_4_H_4_N_2_O_2_	[M + H]^+^	113.0346	113.0346	0.32	96.0079; 70.0287	Uracil (Wang et al., 2024)	O
50	1.79	C_6_H_8_O_7_	[M-H]^-^	191.0186	191.0200	7.02	129.0194; 111.0088; 87.0088	Citric acid (Li et al., 2024c)	OA
51	2.96	C_7_H_10_O_7_	[M-H]^-^	205.0343	205.0356	6.20	143.0357; 111.0087; 87.0086	Trimethyl citrate (Liao et al., 2024)	OA
52	8.65	C_5_H_9_NO_2_	[M + H]^+^	116.0706	116.0704	−1.59	70.0650; 69.0332; 59.0491	Proline (Wang et al., 2024)	OA
53	7.05	C_14_H_18_N_2_O_3_	[M + H]^+^	263.1390	263.1408	6.81	86.0968; 70.0651	Proline-Phenylalanine (Wang et al., 2024)	OA
54	14.27	C_26_H_30_O_13_	[M-H]^-^	549.1603	549.1606	0.68	417.1195; 255.0661; 135.0087	liquiritin apioside (Xu et al., 2021)	F
55	14.13	C_9_H_8_O	[M + H]^+^	133.0648	133.0646	−1.14	115.0539; 105.0698; 103.0540	Cinnamaldehyde (Xiao et al., 2024)	P
56	11.42	C_9_H_8_O_2_	[M + H]^+^	149.0596	149.0596	−0.58	131.0492; 107.0490; 103.0541	Cinnamic acid (Yin et al., 2024)	P
57	24.65	C_9_H_6_O_3_	[M + H]^+^	163.0390	163.0388	−1.23	135.0437; 107.0489; 68.8709	Umbelliferone (Pan et al., 2015)	P
58	13.26	C_15_H_12_O_6_	[M-H]^-^	287.0550	287.0561	3.75	151.0037; 135.0453; 107.0138	Eriodictyol (Xiao et al., 2024)	F
59	11.45	C_27_H_30_O_15_	[M-H]^-^	593.1501	593.1508	1.22	473.1088; 353.0665; 297.0766	Vicenin-2 (Jiang et al., 2024)	F
60	12.26	C_26_H_28_O_14_	[M-H]^-^	563.1395	563.1393	−0.34	383.0769; 353.0664; 297.0765	Schaftoside (Yin et al., 2024)	F
61	4.99	C_8_H_8_O_4_	[M-H]^-^	167.0339	167.0351	7.51	149.0244; 139.0404; 123.0453	Vanillic acid (Yin et al., 2024)	OA
62	13.21	C_9_H_6_O_2_	[M + H]^+^	147.0441	147.0440	−0.31	105.0447	Coumarin (Xiao et al., 2024)	P
63	9.81	C_16_H_18_O_9_	[M-H]^-^	353.0867	353.0870	0.77	191.0562; 179.0349; 135.0453	Neochlorogenic acid (Yang et al., 2024)	P
64	14.49	C_21_H_22_O_9_	[M-H]^-^	417.1180	417.1192	2.93	255.0662; 135.0088	Isoliquiritin (Yin et al., 2024)	F
65	14.58	C_15_H_12_O_4_	[M-H]^-^	255.0652	255.0662	3.90	153.0193; 135.0087	Isoliquiritigenin (Liu et al., 2016)	F
66	8.74	C_15_H_22_N_2_O_4_	[M + H]^+^	295.1652	295.1651	−0.35	136.0755; 86.0964	Isoleucine-tyrosine (Wang et al., 2024)	OA
67	9.58	C_12_H_24_N_2_O_3_	[M + H]^+^	245.1860	245.1859	−0.28	199.1812; 132.1014; 86.0963	Isoleucine-leucine (Wang et al., 2024)	OA
68	4.51	C_10_H_20_N_2_O_4_	[M + H]^+^	233.1496	233.1495	−0.19	86.0963; 74.0600; 72.0809	Isoleucine-threonine (Wang et al., 2024)	OA
69	10.91	C_25_H_24_O_12_	[M-H]^-^	515.1184	515.1199	2.97	353.0878; 191.0561; 179.0351	Isochlorogenic acid A (Yang et al., 2024)	P
70	9.81	C_16_H_18_O_9_	[M-H]^-^	353.0867	353.0870	0.77	191.0562; 179.0349; 135.0453	Cryptochlorogenic acid (MLiu et al., 2023)	P
71	16.28	C_15_H_12_O_5_	[M-H]^-^	271.0601	271.0614	4.69	177.0190; 151.0036	Naringenin (Li et al., 2024b)	F
72	5.91	C_7_H_6_O_4_	[M-H]^-^	153.0182	153.0194	7.55	109.0295	Protocatechuic acid (Liu et al., 2024c)	OA
73	9.61	C_30_H_26_O_12_	[M-H]^-^	577.1341	577.1342	0.19	289.0714	procyanidin B1 (Liu et al., 2023b)	F
74	1.31	C_12_H_22_O_11_	[M-H]^-^	341.1078	341.1086	2.21	179.0561; 161.0455	Sucrose (Wang et al., 2024)	O
75	14.029	C_21_H_20_O_11_	[M-H]^-^	447.0922	447.0931	2.06	285.0394; 284.0317	Astragalin (MLiu et al., 2023)	F

Note: F = flavonoids; OA, organic acids; P = phenylpropanoids; T = terpenoids; S = saponins; O = others.

### 3.3 Prediction active components of LGZGD in UA treatment

#### 3.3.1 Compound-disease intersection targets

**FIGURE 2:**
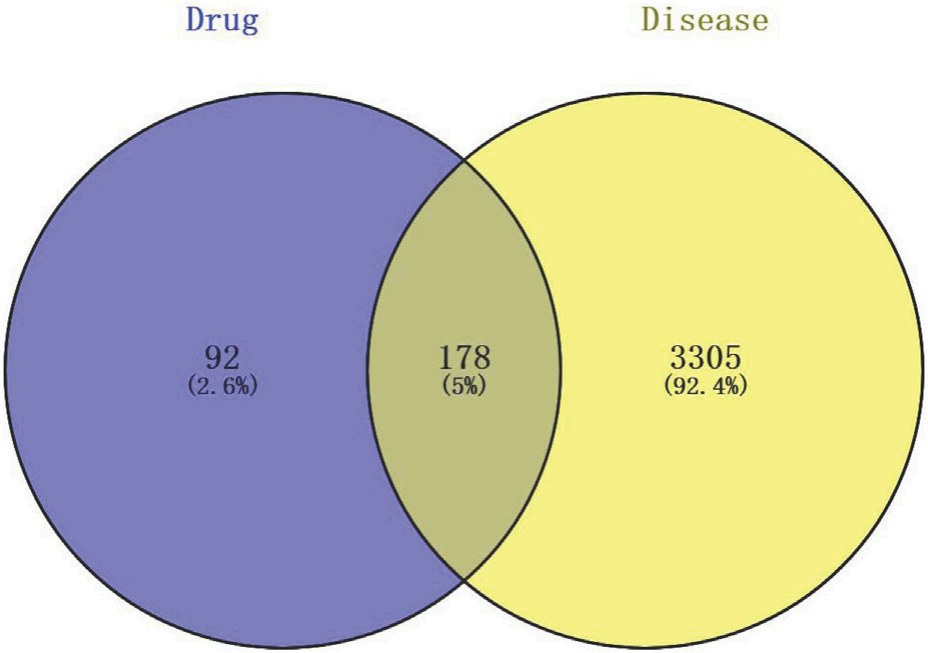
Venn diagram of LGZGD compound and UA disease target overlap.

From the screening process, 11 compounds with oral bioavailability (OB) > 30% and drug-likeness (DL) > 0.15 were selected. A total of 270 unique targets were predicted for these compounds. Simultaneously, a disease-target database search using the term “unstable angina” across OMIM and GeneCards yielded 3,483 human UA-related targets after deduplication. By intersecting the compound and disease targets, 178 overlapping targets were identified (Figure 2).

#### 3.3.2 PPI network analysis

**FIGURE 3:**
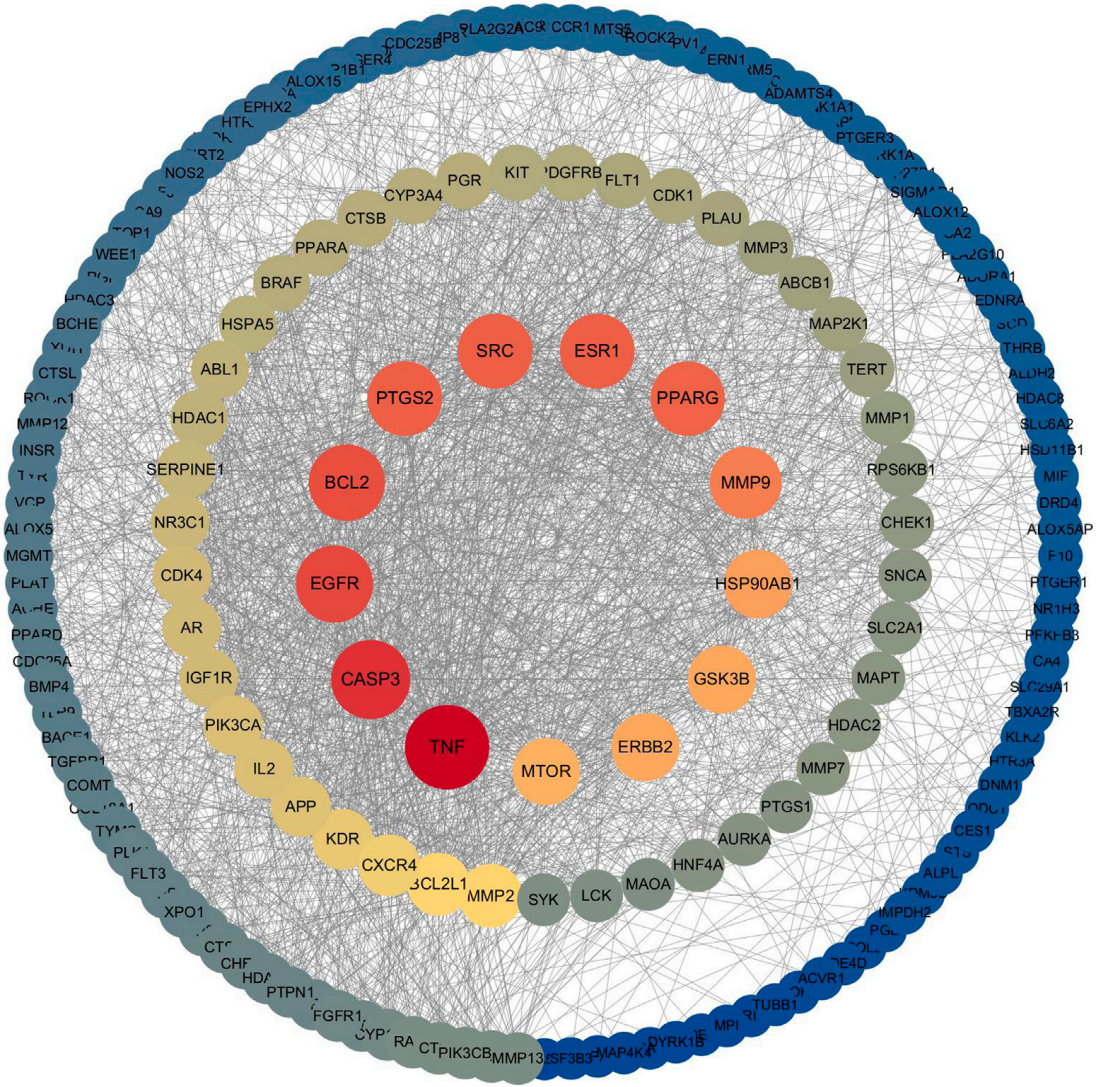
PPI network of overlapping compound-disease targets.

**FIGURE 4:**
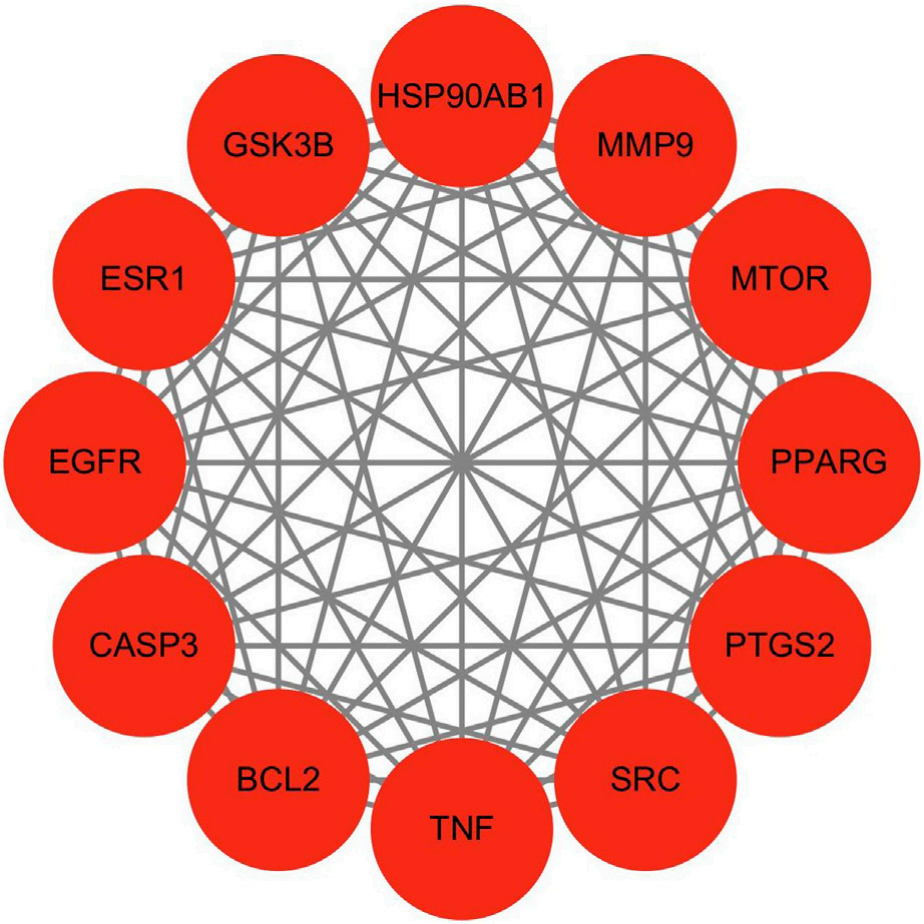
Core target network identified by CytoNCA analysis.

The 178 overlapping targets were submitted to the STRING v12.0 database, and a protein-protein interaction (PPI) network was constructed using Cytoscape. Node color and size in Figure 3 represent interaction intensity: red indicates high connectivity, and blue indicates low. Core target analysis using the CytoNCA plug-in identified 12 hub proteins, shown in Figure 4. Among these, the top five by degree centrality were Tumor Necrosis Factor (TNF), Caspase-3 (CASP3), Epidermal Growth Factor Receptor (EGFR), BCL2 Apoptosis Regulator (BCL2), and Prostaglandin-Endoperoxide Synthase 2 (PTGS2). These targets are highly interconnected and are likely pivotal in mediating LGZGD’s therapeutic effects against UA. They were thus selected for molecular docking validation.

#### 3.3.3 GO and KEGG enrichment analysis

**FIGURE 5:**
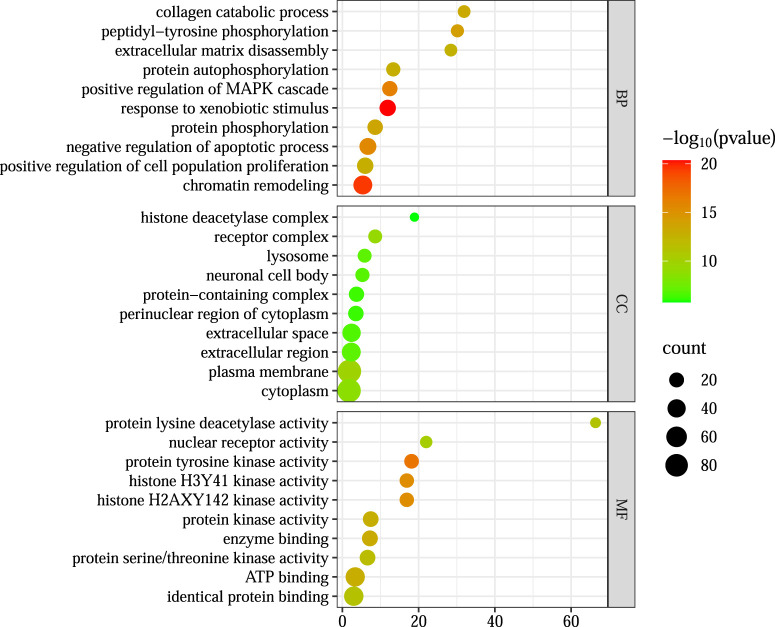
GO enrichment analysis.

**FIGURE 6:**
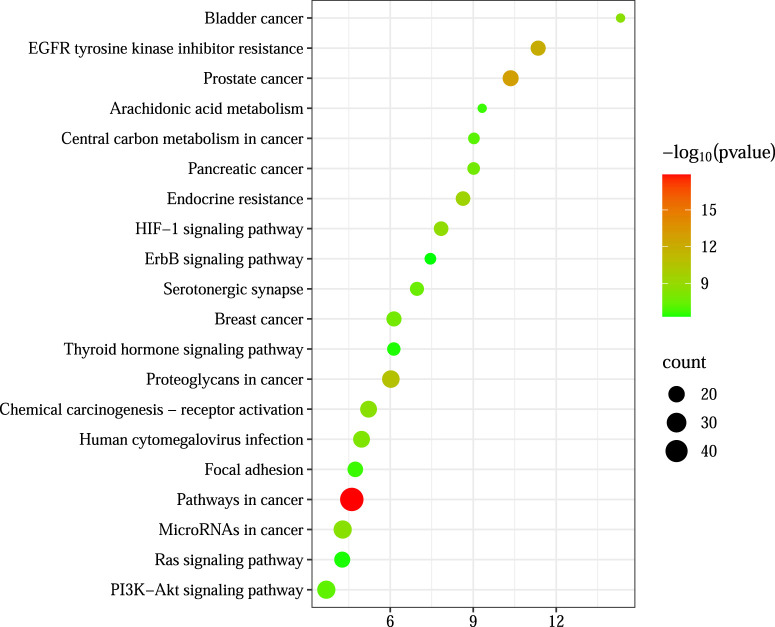
KEGG enrichment analysis.

Gene Ontology (GO) and Kyoto Encyclopedia of Genes and Genomes (KEGG) enrichment analyses were performed on the 178 intersecting targets using the DAVID v6.8 database. The GO analysis yielded 633 biological processes (BP), 93 cellular components (CC), and 192 molecular functions (MF), while KEGG pathway analysis identified 139 signaling pathways. The top 10 enriched GO terms and the top 20 KEGG pathways, ranked by *p*-value, are visualized in Figures 5, 6 In these plots, the y-axis represents the enriched terms or pathways, and the x-axis indicates the enrichment ratio. Larger node sizes denote a greater number of enriched genes, darker node colors indicate higher statistical significance, and points further to the right reflect stronger enrichment correlations.

Based on these enrichment results, the primary biological processes involved in LGZGD’s therapeutic effects against UA appear to include responses to xenobiotic stimuli and chromatin remodeling. Key cellular components include the plasma membrane and cytoplasm. Relevant molecular functions are associated with protein lysine deacetylase activity, histone H3Y41 kinase activity, and histone H2AXY142 kinase activity.

#### 3.3.4 Construction of the “medicine-component-target-pathway-disease” network model

**FIGURE 7:**
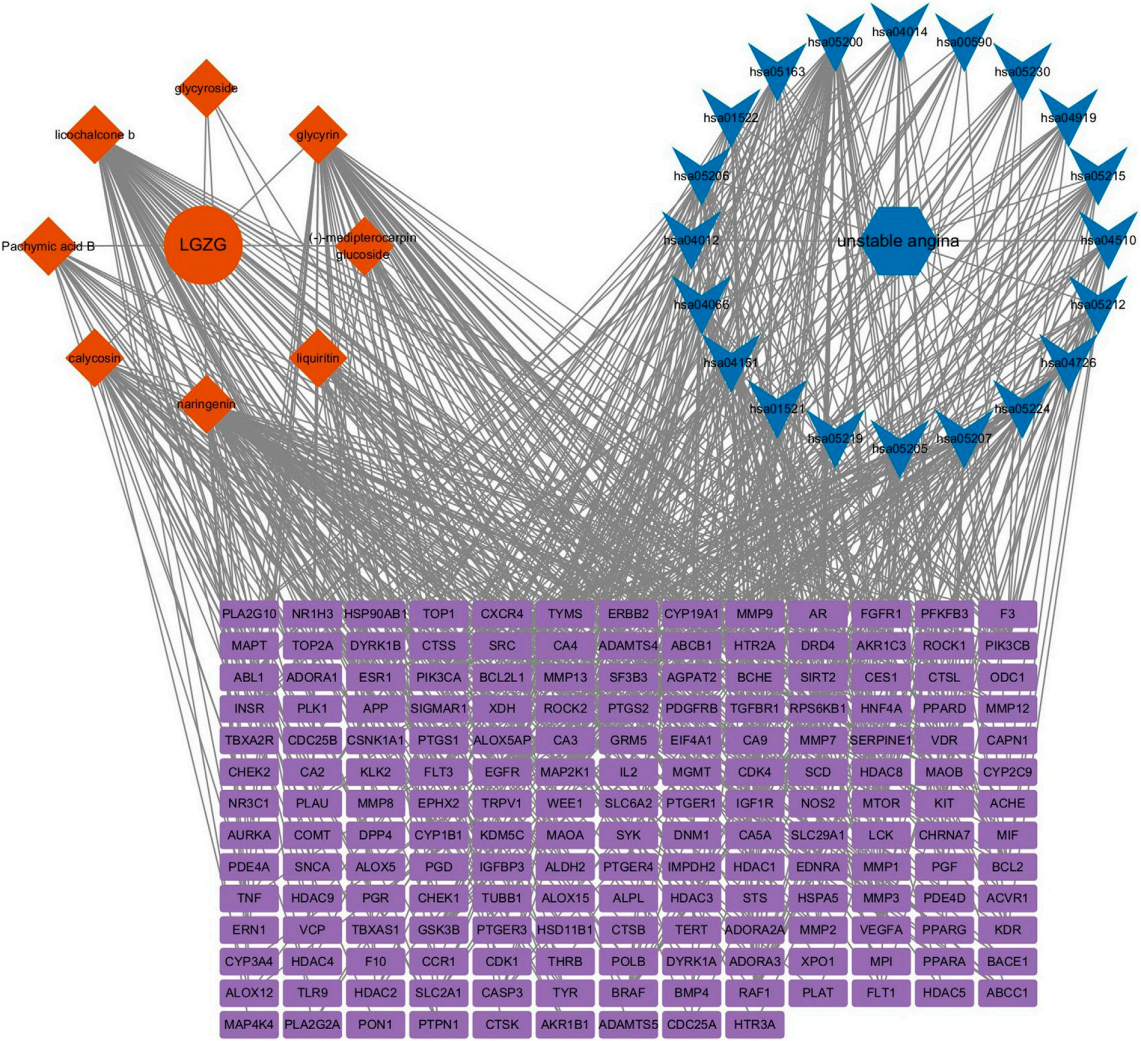
Drug-component-target-pathway-disease network model.

A comprehensive “medicine–component–target–pathway–disease” interaction network was constructed and is illustrated in Figure 7. Based on degree centrality values, naringenin, licochalcone B, glycyrin, calycosin, and pachymic acid B were identified as key active compounds likely responsible for LGZGD’s therapeutic effects in UA.

#### 3.3.5 Molecular docking verification

**FIGURE 8:**
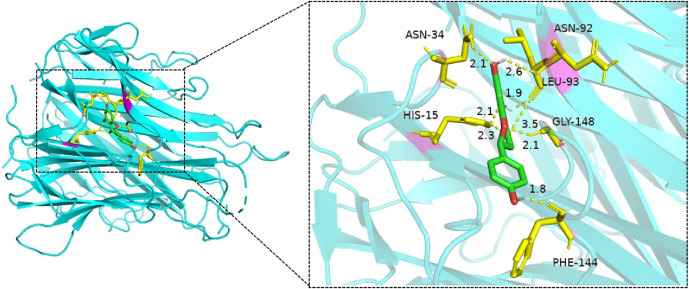
Molecular docking of naringenin with TNF.

**FIGURE 9:**
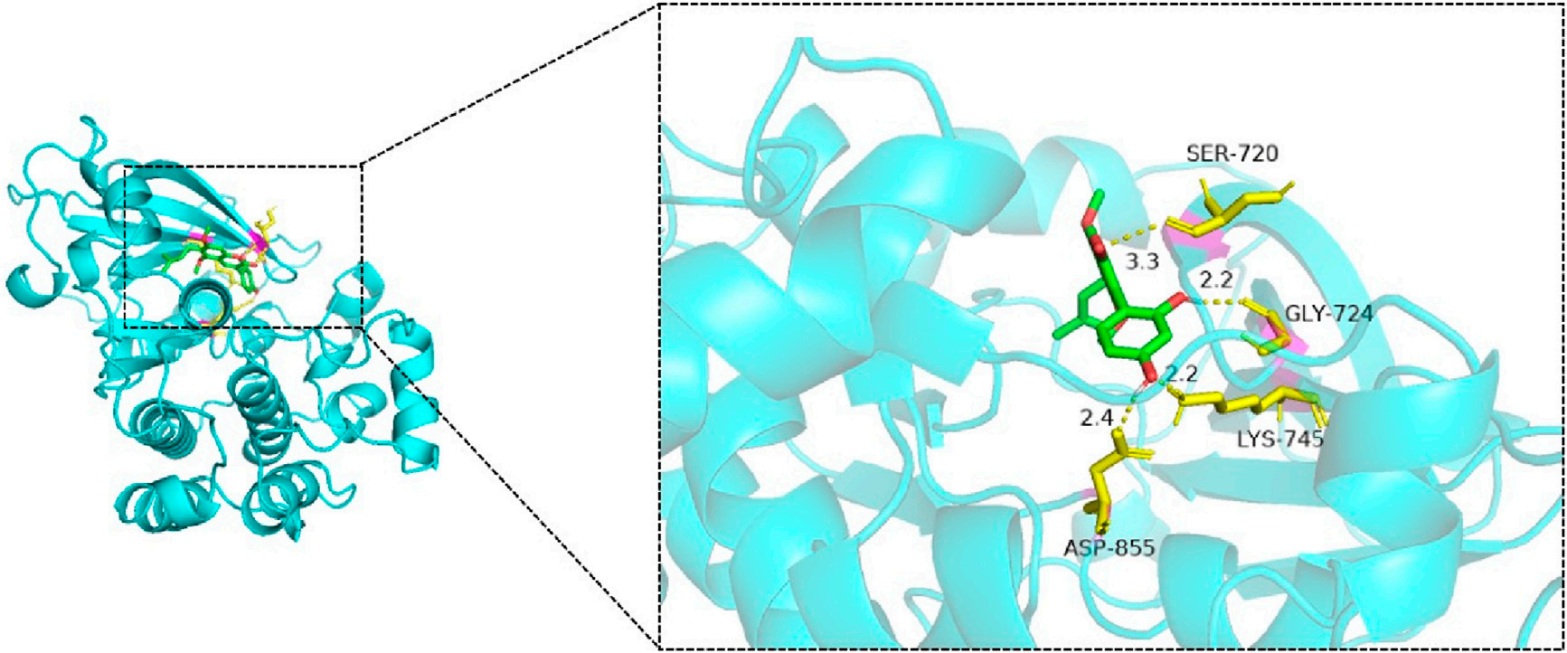
Molecular docking of glycyrrhizin with EGFR.

**FIGURE 10:**
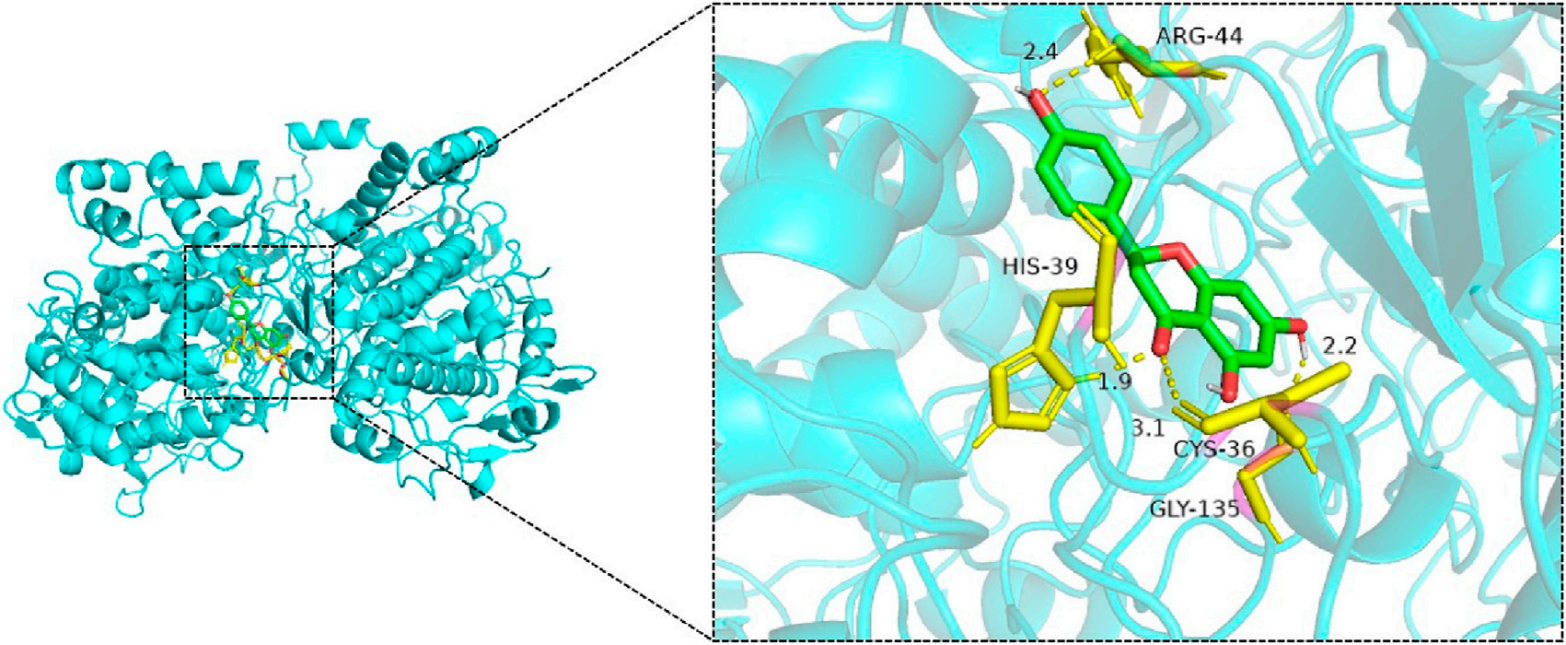
Molecular docking of naringenin with PTGS2.

**FIGURE 11:**
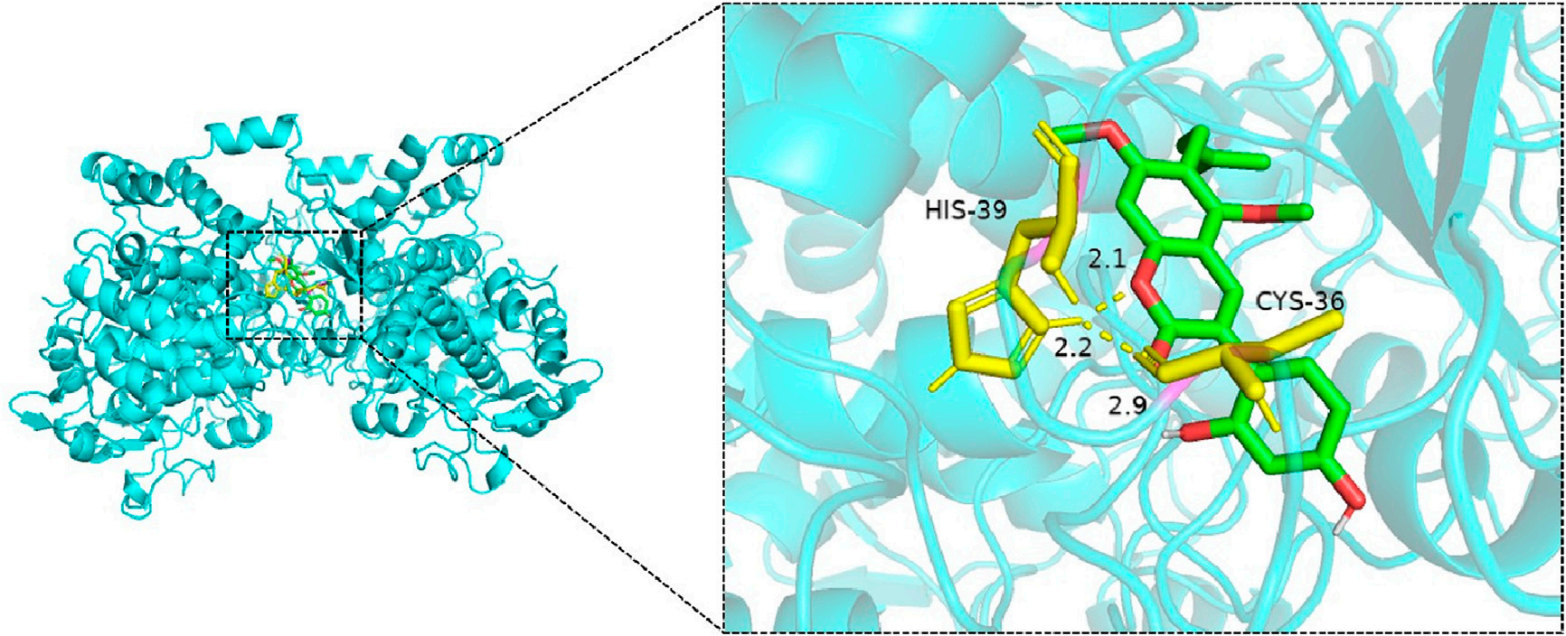
Molecular docking of glycyrrhizin with PTGS2.

**FIGURE 12:**
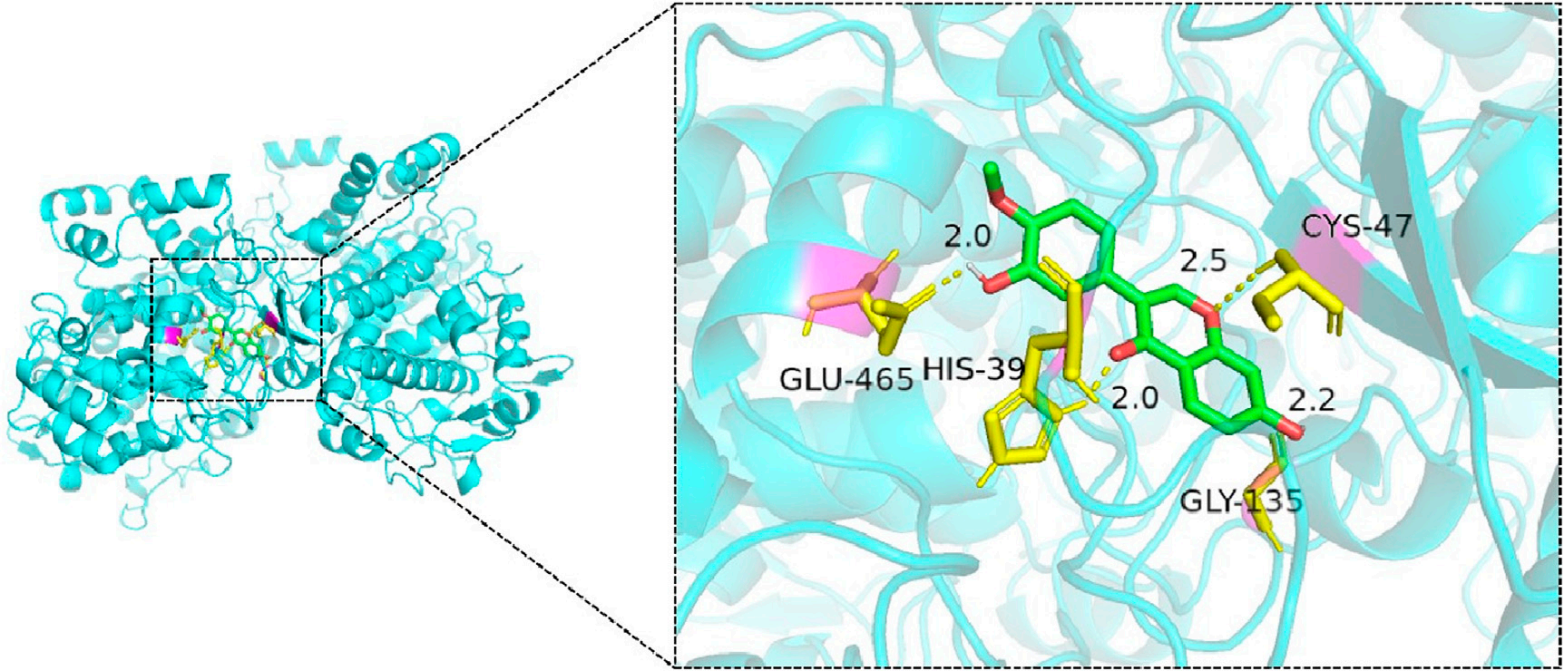
Molecular docking of calycosin with PTGS2.

Molecular docking simulations were performed for 25 ligand–receptor pairs. All combinations exhibited binding energies below zero, indicating spontaneous binding. Five combinations showed binding energies lower than −5.0 kcal/mol, signifying good binding affinity, while energies below −7.0 kcal/mol were considered indicative of strong interactions. These results are shown in Figures 8–12. The most stable docking interaction was between naringenin and TNF, with a binding energy of −8.03 kcal/mol. Notably, PTGS2 demonstrated consistently strong binding with multiple ligands: naringenin, glycyrrhizin, and calycosin all exhibited binding energies below −5.0 kcal/mol with this target.

## 4 Discussion

### 4.1 Optimization of preliminary experimental conditions

In the concentration step, vacuum concentration was employed instead of atmospheric pressure concentration to minimize the loss of volatile compounds, considering that Guizhi is rich in low molecular weight volatile substances such as alkenes, aldehydes, phenols, and alcohols. Fuling primarily contains polysaccharides, particularly alkali-soluble polysaccharides, which constitute 70%–90% of the total polysaccharide content. These alkali-soluble components are poorly water-soluble and thus challenging to extract via traditional decoction method. Furthermore, previous studies indicate that key triterpenes in Fuling, such as pachymic acid, dehydrotumulosic acid, and polyporus acid C, are inefficiently extracted by decoction. Additionally, the presence of 5-hydroxymethylfurfural in *Atractylodes macrocephala* and *Glycyrrhiza uralensis* can interfere with analysis.

Through preliminary experimentation, we determined that neochlorogenic acid and cryptochlorogenic acid in *A. macrocephala* met the quantification criteria and did not exhibit negative interference with components in Fuling, Ramulus cinnamomi, or *G. uralensis*. Based on these findings, the following compounds were selected as marker components for transfer rate studies: glycyrrhizin and glycyrrhizic acid from *G. uralensis*; cinnamaldehyde and cinnamic acid from *Ramulus cinnamomi*; and neochlorogenic acid and cryptochlorogenic acid from *A. macrocephala*.

### 4.2 Fragmentation pathways of LGZGD

#### 4.2.1 Flavonoids

Flavonoids are polyphenolic secondary metabolites commonly found in plants and diets. They share a characteristic C6–C3–C6 skeleton, with two aromatic rings connected by a three-carbon bridge. Flavonoids typically undergo Retro Diels–Alder (RDA) fragmentation, involving the loss of CO, CO_2_, and H_2_O.

In negative ion mode, compound 27 produced a deprotonated molecular ion at *m/z* 417.1195 [M–H]^-^, consistent with a molecular formula of C_21_H_22_O_9_. Fragment ions at *m/z* 255.0662 and *m/z* 135.0088 corresponded to the successive loss of a glucose moiety (C_6_H_10_O_5_) and another sugar-derived fragment (C_14_H_18_O_6_), respectively. Based on these characteristics, the compound was identified as liquiritin, and its MS^2^ spectrum and fragmentation pathway are shown in Supplementary Figure S3.

In positive ion mode, compound 48 showed a protonated molecular ion at *m/z* 285.0760 [M + H]^+^, corresponding to C_16_H_12_O_5_. Fragment ions at *m/z* 270.0524 [M + H–CH_3_]^+^, 253.0496 [M + H–CH_4_O]^+^, and 225.0545 [M + H–C_2_H_4_O_2_]^+^ were consistent with successive losses of a methyl group, a hydroxyl group, and a carbonyl group. This fragmentation pattern confirmed the compound as calycosin. Its MS^2^ spectrum and fragmentation pathway are illustrated in Supplementary Figure S4.

#### 4.2.2 Organic acids

Organic acids are characterized by the presence of acidic functional groups such as carboxyl (-COOH), sulfonic acid (-SO_3_H), sulfinic acid (-SOOH), and thiocarboxylic acid (-SH). This category includes organic acids, phenols, phenolic acids, and their esters. These compounds typically exhibit simple structures and ionize efficiently in negative ion mode. Fragmentation commonly involves neutral losses of CO_2_ and H_2_O. In negative ion mode, compound 43 exhibited a quasi-molecular ion peak at *m/z* 191.0559 [M–H]^-^, corresponding to the molecular formula C_7_H_12_O_6_. Secondary mass spectrometry revealed fragment ions at *m/z* 129.0200 [M–H–CO_2_–H_2_O]^-^ and *m/z* 111.0088 [M–H–5O]^-^. Based on this data, the compound was identified as quinic acid, with its fragmentation pattern illustrated in Supplementary Figure S5.

#### 4.2.3 Phenylpropanoids

Phenylpropanoids consist of a benzene ring connected to a three-carbon side chain (C_6_–C_3_) and often possess phenolic structures. They include phenylpropanoic acids, coumarins, and lignans. Due to the presence of ester groups, these compounds are prone to fragmentation, particularly via loss of CO_2_ from carboxyl groups. In negative ion mode, compound 63 displayed a quasi-molecular ion peak at *m/z* 353.0870 [M–H]^-^, suggesting a molecular formula of C_16_H_18_O_9_. Fragment ions at *m/z* 179.0349 [M–H–C_7_H_10_O_5_]^-^ and *m/z* 135.0453 [M–H–C_7_H_10_O_5_–CO_2_]^-^ indicated cleavage of an ester group and subsequent CO_2_ loss. The compound was identified as neochlorogenic acid, with its fragmentation profile shown in Supplementary Figure S6.

In positive ion mode, compound 56 exhibited a quasi-molecular ion peak at *m/z* 149.0596 [M + H]^+^, corresponding to C_9_H_8_O_2_. Its MS^2^ spectrum showed fragment ions at *m/z* 131.0492 [M + H–H_2_O]^+^ and *m/z* 103.0541 [M + H–H_2_O–CO]^+^. This compound was identified as cinnamic acid, and its fragmentation pathway is shown in Supplementary Figure S7.

#### 4.2.4 Terpenes

Terpenoids are composed of isoprene units linked in various configurations and include monoterpenes, sesquiterpenes, and triterpenes. In positive ion mode, compound 11 displayed a quasi-molecular ion peak at *m/z* 231.1379 [M + H]^+^, with a deduced molecular formula of C_15_H_18_O_2_. MS^2^ analysis revealed fragment ions at *m/z* 203.0854 [M + H–CO]^+^, *m/z* 163.0753 [M + H–C_5_H_8_]^+^, and *m/z* 119.0855 [M + H–C_5_H_8_–CO_2_]^+^. This pattern is consistent with atractylenolide I, and its cleavage pathway is presented in Supplementary Figure S8.

#### 4.2.5 Saponins

Saponins are glycosides formed by the linkage of sapogenins and sugar moieties through glycosidic bonds. In negative ion mode, compound 31 showed a quasi-molecular ion peak at *m/z* 821.3957 [M–H]^-^, corresponding to C_42_H_62_O_16_. MS^2^ analysis revealed fragment ions at *m/z* 803.3861 [M–H–H_2_O]^-^, *m/z* 759.3911 [M–H–H_2_O–CO_2_]^-^, and *m/z* 645.3616 [M–H–C_6_H_8_O_6_]^-^. These fragments supported the identification of glycyrrhizic acid, with its cleavage pattern shown in Supplementary Figure S9.

### 4.3 Changes in LGZGD chemical composition after spray drying

Five compounds were not detected in LGZGD after spray drying: (–)-mediphyllin glucoside, γ-aminobutyric acid, calycosin, trimethyl citrate, and proline-phenylalanine. (-)- medipterocarpin glucoside, a bioactive component of the traditional Chinese formulation Wendan decoction, exhibits high oral bioavailability and favorable drug-like properties. γ-Aminobutyric acid (GABA), a key inhibitory neurotransmitter, is implicated in the regulation of sleep-wake cycles; disruptions in the GABA/glutamate balance are central to the pathophysiology of sleep disorder. Calycosin and its derivatives exhibit anti-inflammatory, anti-apoptotic, and antitumor effects. Notably, calycosin protects the cardiovascular system by mitigating myocardial ischemia and hypoxia and improving cardiomyocyte surviva. Trimethyl citrate is a biodegradable pharmaceutical intermediate widely used across medical, food, and chemical industries. Although proline-phenylalanine was undetectable post-drying, its constituent amino acids, proline and phenylalanine, were still present, suggesting peptide bond hydrolysis under high temperatures. These findings indicate that spray drying may degrade thermolabile bioactive compounds. To minimize such losses, alternative low-temperature drying techniques, such as vacuum freeze-drying, should be considered during the formulation of LGZGD solid preparations.

### 4.4 Molecular docking verification

Animal studies by (Ren et al., 2024) demonstrated that LGZGD significantly improved cardiac function and reduced myocardial histopathological damage in mice with chronic heart failure following myocardial infarction. This effect was linked to the activation of the HIF-1α/HO-1 signaling pathway and autophagy. Through network pharmacology, quercetin and naringenin were identified as core active compounds targeting HO-1 and HIF-1α, both of which displayed low binding energies. Naringenin was confirmed in the current LC-MS analysis, suggesting it may play a crucial role in LGZGD’s cardioprotective mechanisms.

In related work, (Zhao et al., 2023) utilized network pharmacology to investigate LGZGD’s effects on coronary heart disease. Key compounds included quercetin, kaempferol, naringenin, isorhamnetin, and formononetin, with core targets such as AKT1, TP53, STAT3, IL-6, and EGFR. Molecular docking confirmed strong binding interactions (binding energies <−5 kcal/mol) between these compounds and their respective targets, reinforcing their potential pharmacological relevance.

## 5 Conclusion

In this study, UHPLC-Q-Orbitrap/MS technology was employed for the first time to characterize the chemical constituents of LGZGD intermediates. A total of 75 compounds were identified, among which five were absent following spray drying, suggesting potential loss of efficacy due to processing. Network pharmacology analysis, combined with molecular docking, was also employed—again, for the first time—to predict and validate the pharmacodynamic material basis of LGZGD in the treatment of UA. This analysis identified three core active components: naringenin, licorice-derived constituents, and calycosin. Notably, calycosin was one of the five components lost during spray drying, implying that this preparation method may influence the therapeutic efficacy of LGZGD.

Subsequent HPLC analysis confirmed the presence of naringenin and calycosin in the extract, with signal-to-noise ratios of 4 and 8—above the detection limit but below the quantification threshold. Therefore, more precise quantification may require the use of liquid chromatography–mass spectrometry. Additionally, key index compounds in LGZGD—including cinnamic acid, cinnamaldehyde, neochlorogenic acid, cryptochlorogenic acid, liquiritin, and glycyrrhizic acid—were quantitatively analyzed for the first time. Their transfer rates across decoction pieces and various preparation intermediates were also elucidated.

Despite these advancements, the scope of the current study remains limited. Future work should incorporate animal experiments and explore additional carriers to better establish the pharmacodynamic basis of classical prescriptions, support the development of compound formulations, and guide rational clinical application.

## Data Availability

The original contributions presented in the study are included in the article/Supplementary Material, further inquiries can be directed to the corresponding author.
