# Supplementary material for: Predict potential pharmacological mechanisms of Ling-gui-Zhu-gan Decoction in treating unstable angina pectoris using liquid chromatography-mass spectrometry and network pharmacology
Source: Front Chem. 2025 Aug 4;13:1649538. doi: 10.3389/fchem.2025.1649538 (PMC12358372; doi:10.3389/fchem.2025.1649538)
Supplement: Supplementary file 1 [file DataSheet1.pdf]

supplementary materials

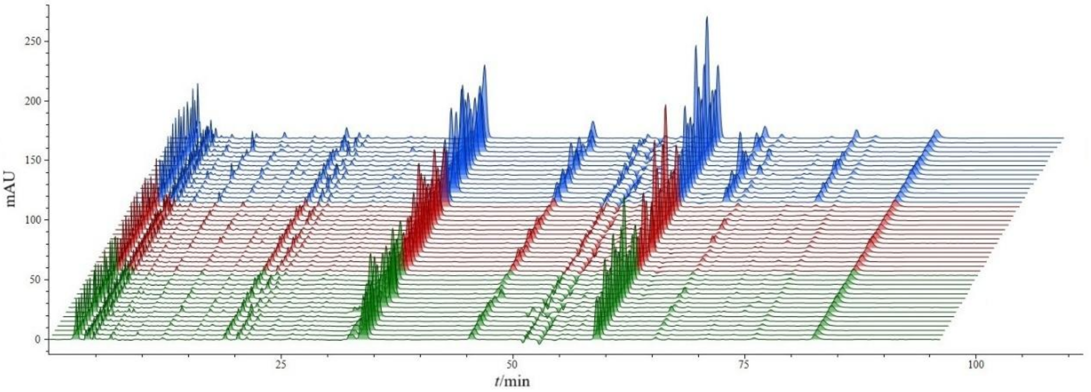

Supplementary Fig.1 Overlay chromatograms of LGZGD samples.

Note: Blue, red, and green lines represent decoction pieces (WD), concentrated solution (CS), and spray-dried powder (SDP), respectively.

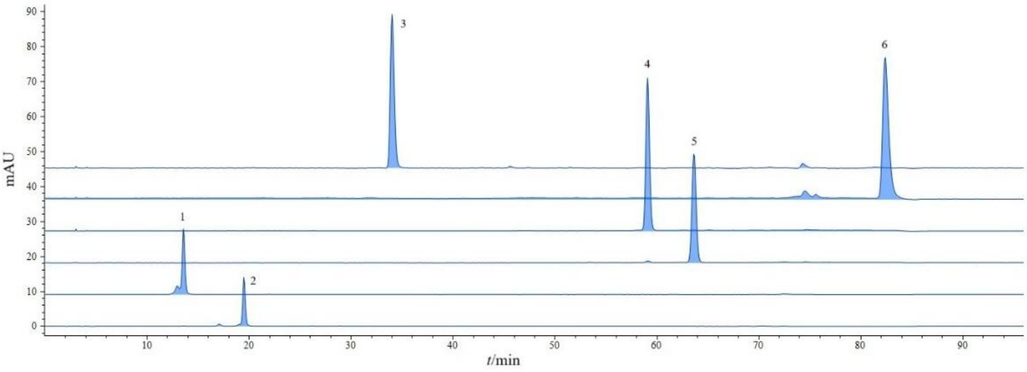

Supplementary Fig.2 Overlay chromatogram of LGZGD reference standards.

Note: Peak 1: neochlorogenic acid; Peak 2: cryptochlorogenic acid; Peak 3: liquiritin; Peak 4: glycyrrhizic acid; Peak 5: cinnamaldehyde; Peak 6: cinnamic acid.

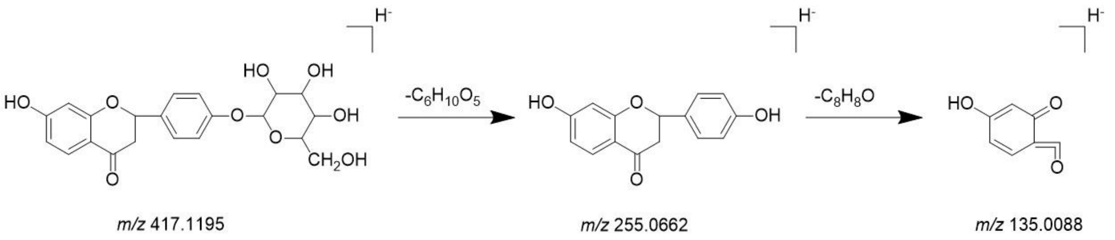

Supplementary Fig.3 MS/MS spectrum and proposed fragmentation pathway of liquiritin.

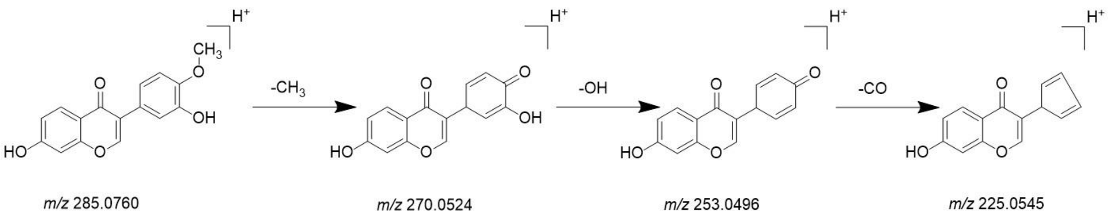

Supplementary Fig.4 MS/MS spectrum and proposed fragmentation pathway of calycosin.

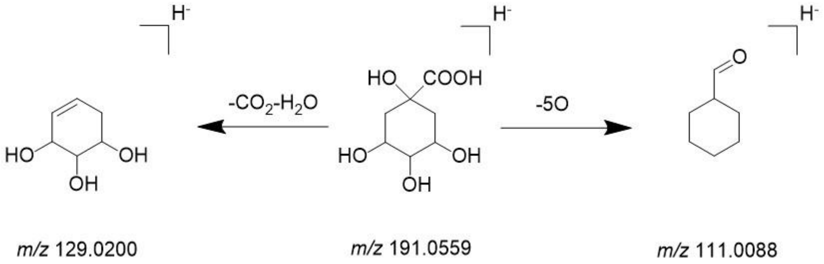

Supplementary Fig.5 Cleavage pathway of quinic acid.

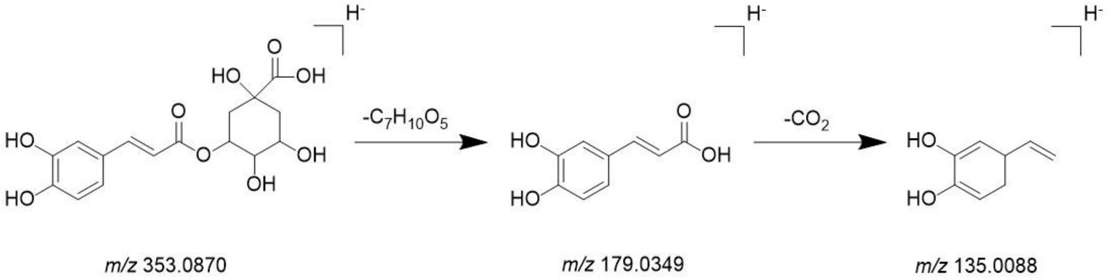

Supplementary Fig.6 Cleavage pathway of neochlorogenic acid.

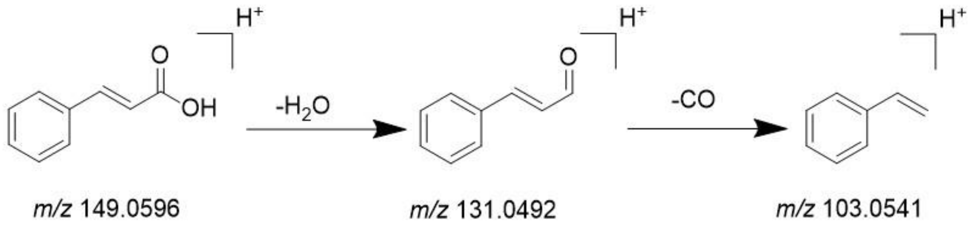

Supplementary Fig.7 Cleavage pathway of cinnamic acid.

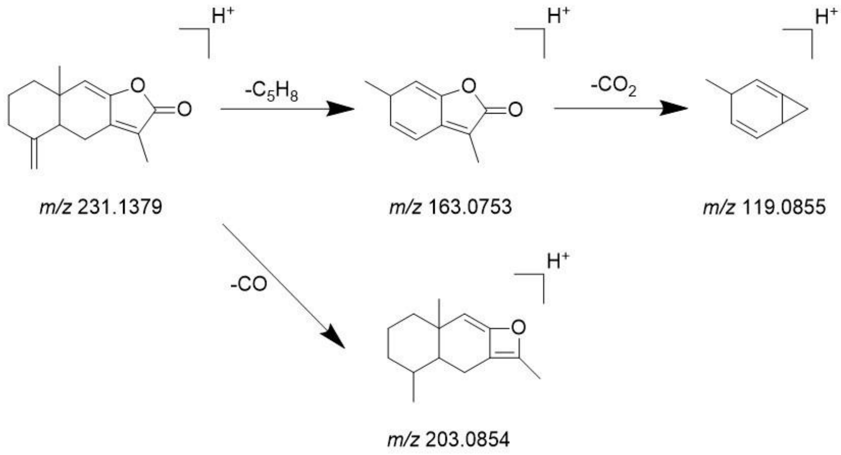

Supplementary Fig.8 Cleavage pathway of Atractylenolide I.

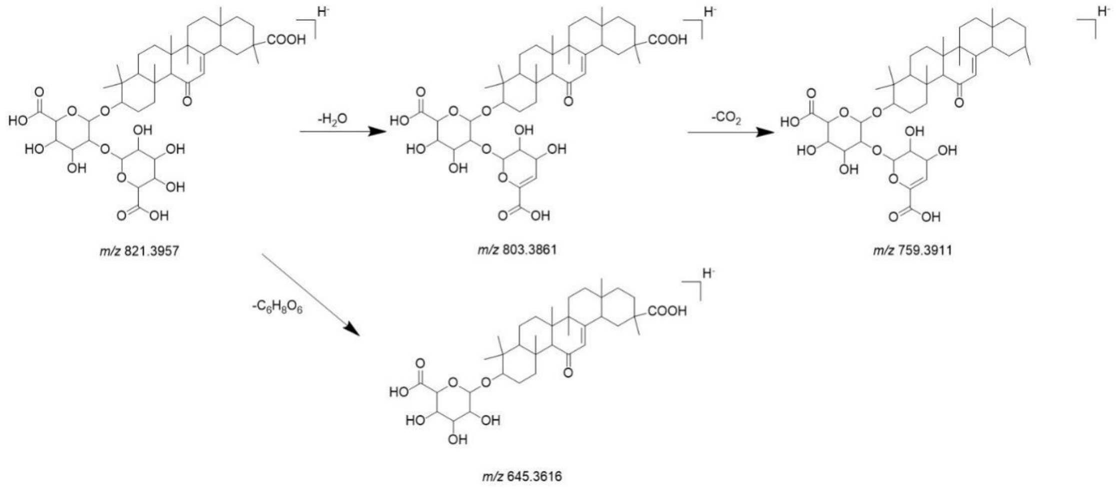

Supplementary Fig.9 Cleavage pathway of glycyrrhizic acid.

Supplementary Table 1. Linear calibration results for marker compounds.

Index Component	Regression Equation	Linear Range (μg·mL^{-1}).	R^2
Liquiritin	Y = 13.942 X + 27.341	8.28~289.80	0.9993
Glycyrrhizic acid	Y = 5.8952 X - 3.0105	28.21~620.71	0.9997
Cinnamic acid	Y = 52.825 X + 68.723	1.90~95.15	0.9993
Cinnamaldehyde	Y = 102.05 X - 31.428	0.05~36.39	0.9990
Neochlorogenic acid	Y = 11.701 X - 13.592	0.63~62.6	0.9992
Cryptochlorogenic acid	Y = 9.7809 X - 0.461	13.96~1395.98	0.9993

Supplementary Table 2. Precision, repeatability, stability, and recovery (RSD of peak areas).

Index Component	Precision RSD	Repeatabilit RSD	Stability RSD	Sample Recovery	Sample Recovery RSD
Liquiritin	0.92%	1.37%	2.54%	106.27%	1.50%
Glycyrrhizic acid	0.98%	0.98%	1.25%	93.01%	1.94%
Cinnamic acid	0.61%	0.79%	0.89%	107.31%	1.45%
Cinnamaldehyde	0.37%	1.52%	0.94%	92.23%	1.70%
Neochlorogenic acid	3.50%	1.58%	2.80%	96.52%	2.36%
Cryptochlorogenic acid	1.29%	1.29%	2.22%	93.33%	2.28%
